# Comparative study of antipyretic potency of extracts of morinda lucida leaves and fruits of capsicum frutescens in albino rats

**DOI:** 10.4314/ahs.v23i1.23

**Published:** 2023-03

**Authors:** Adedotun A Adefolalu, Bamidele V Owoyele, Ayoade A Adesokan

**Affiliations:** 1 Department of Biochemistry, P.M.B 146, Federal University Lafia, Nigeria; 2 Department of Physiology, College of Medicine, University of Ilorin, Nigeria; 3 Department of Biochemistry, University of Ilorin, Nigeria

**Keywords:** *Morinda lucida*, *Capsicum frutescens*, brewer's yeast, antipyretic, rectal temperature

## Abstract

**Background:**

*Morinda lucida* leaves and fruits of *Capsicum frutescens* are used locally in the management of fever in Nigeria. No scientific credence has been lent to this claim.

**Objective:**

To investigate the antipyretic effect and potency of aqueous extracts of *Morinda lucida* leaves and fruits of *Capsicum frutescens* in albino rats.

**Method:**

Brewer's yeast was used to induce pyrexia. Thirty animals were divided into six groups. Group A was orally administered normal saline (103 mg/kg). Group B was served indomethacin (5 mg/kg), while groups C and D received aqueous extract of *Capsicum frutescens* at 100mg/kg and 200mg/kg, 17 hours post induction of pyrexia. Groups E and F were administered extract of *Morinda lucida* at the same doses. Rectal temperature of the animals was taken at 60-, 90- and 120-minutes post-treatment.

**Results:**

Both *C. frutescens* and *M. lucida* produced significant reduction (p<0.05) in rectal temperature after 120 minutes in the rats compared with animals in the control group. Also, the antipyretic activities of the two extracts at 100mg/kg and 200mg/kg were comparable to 5mg/kg of indomethacin, with apparent dose dependence in the antipyretic activities of both extracts.

**Conclusion:**

*Morinda lucida* leaves and fruits of *Capsicum frutescens* exhibit dose-dependent antipyretic activities.

## Introduction

William Osler 1 famously posits that “humanity has but three great enemies: fever, famine, and war; of these by far the greatest and the most terrible is fever.” Fever is the oldest physiological response to disease and infection, characterized by elevated body temperature. While fever usually occurs in response to infections and inflammations, it often also occurs as an immuno-adaptive response, which is either infectious or non-infectious 2. To reconcile these adaptations, the International Union of Physiological Sciences defines fever as “a state of elevated core temperature, which is often, but not necessarily, part of the defensive responses of multicellular organisms (host) to the invasion of live (micro-organisms) or inanimate matter recognised as pathogenic or alien by the host” 3. Clinicians have managed and treated febrile response due to different diseases by various means. However, the prominence of the so-called ‘fever of unknown origin’, defined by Petersdorf and Beeson 4 as “fever higher than 38.3°C which persists without diagnosis for at least three weeks despite at least one week's investigation in a hospital”, makes it imperative to identify novel anti-pyretic agents.

Many products derived from plants have been used for medicinal purposes for centuries. It is estimated that about 80% of the world population, presently relies on botanical preparations as medicines to meet their health needs 5. In Nigeria, various plants and plant parts are used in treating diseases such as fever, dysentery, sore throat, skin diseases etc. Some of these therapies have been confirmed and backed by scientific research 6–8. *Morinda lucida* is used traditionally as antimalarial, as well as anti-pyrexia^9^. Its green leaves are used as an ingredient of “fever teas”, which are usually taken for the traditional treatment of fever and malaria 10. Many investigations have studied the therapeutic benefits of the plant 11–15, attributed to its constituent secondary metabolites 16–17. While leaf extract of *Morinda lucida* has been investigated for various therapeutic benefits as claimed by traditional users 18, its antipyretic activity has not been ascertained.

*Capsicum frutescens* is believed traditionally to possess antimalarial and antipyretic properties, and is usually used in decoctions, commonly called ’agbo’ in south western Nigeria, where it is called ‘ata-ijosi 9. *Capsicum* is used and loved the world over as a condiment, added to food, fresh, dried, refined, and ground (for Cayenne pepper and curry), and as the principal or incidental ingredient in sauces^19^. The source of the popular pungent, biting sensation in *C. frutescens* are the capsaicinoids, alkaloid compounds that are found only in the plant genus, *Capsicum*, often called capsaicin after the most prevalent one 20.

Many studies have been carried out to investigate possible medicinal uses of capsaicin. It was shown that capsaicin has antibacterial activities against Helicobacter pylori 21–22. In Nigeria, *Capsicum frutescens* is used either solely, or in combination with other herbs as decoctions in treating various diseases, especially malaria 9; this is despite its strong biting sensation. The active principle of these herbs is often extracted through infusion, decoction and tincture 23. This study investigates the potential antipyretic properties of this fruit, and compares its potency with another commonly used traditional herb, *Morinda lucida*.

## Materials and Methods

### Experimental animals

Thirty white albino rats (*Rattus novergicus*) were used for this study. The animals were obtained from the animal house of the Department of Biochemistry, University of Ilorin. They were housed in metabolic cages under standard conditions (25-29°C, 12-hour light and 12-hour darkness cycles). They were fed rat chow (Bendel Feeds and Flour mills Ltd., Ewu, Nigeria) and given water *ad libitum*. The study was conducted in accordance with the ethics of animal experimentation of the University of Ilorin, Nigeria, which represents international principles on animal experimentation.

### Plants

Fresh fruits of *C. frutescens* and leaves of *M. lucida* were obtained from herb sellers in ‘Oja Oba’ market in Ilorin metropolis, Kwara state, Nigeria. They were air-dried at room temperature in the laboratory, Biochemistry Department, University of Ilorin.

### Reagents and drugs

Indomethacin, from Tuyil Pharmacy was used as the reference drug, while BioFlex® normal saline (0.9% NaCl) from Momrota Pharmacy, Ilorin was administered to the control group. Brewer's yeast, a well-documented fever inducing agent 24–26 was used to induce hyperpyrexia. It was obtained from Sona breweries, Sango-Ota, Ogun state, Nigeria. Distilled water, obtained from the Department of Chemistry, University of Ilorin was used to constitute the extracts, as well as indomethacin, the reference drug into doses.

### Extraction procedures

The fresh *M. lucida* leaves and fruits of *C. frutescens* were air-dried until a constant weight resulted. The dried samples were ground, and 100g of each was percolated, each into 1000 litres of distilled water for 48 hours. Whatmann no. 1 filter paper was used to separate both mixtures. The *M. lucida* filtrate gave a brown colour, while that of *C. frutescens* was faint yellow in colour. The filtrates were evaporated to a constant weight, on a carefully regulated water bath, at 40°C. The resultant extracts were preserved in a refrigerator at 4°C.

### Experimental design

Thirty rats were randomly distributed into six groups (A-F) of five animals each, with a female animal in each group. The female rats were kept in a separate cage to forestall any incidence of coition. The animals were acclimatized for one week, and allowed free access to rat chow and water, *ad libitum*. Group A, the control, was orally administered l0^3 mg/kg of normal saline and the positive control was administered 5 mg/kg of indomethacin. Two test groups were orally administered aqueous leaf extract of *Morinda lucida* at the doses of 100 mg/kg and 200 mg/kg body weight. The remaining two groups were similarly administered aqueous extract of *Capsicum frutescens* at the same doses of 100 mg/kg and 200 mg/kg.

### Antipyretic study

The method adopted was as previously described 6. Brewer's yeast was used to induce pyrexia. The animals used were deprived of food, the night preceding the injection of brewer's yeast, while making water available, *ad libitum*^6^. Rectal temperatures of the animals were taken before induction of pyrexia, using a clinical thermometer probe, inserted 2-3cm into the rectum of the animals. Pyrexia was induced by subcutaneous injection of 20% (w/v) of brewer's yeast suspension at a dose of 10 mg/kg into the back of the rats, near the groin. The rectal temperature was again measured, 17 hours post induction of pyrexia, after which the animals were treated with normal saline, indomethacin and the different doses of the extract accordingly. The rectal temperature of each rat was subsequently measured at 60-, 90- and 120-minutes post-treatment.

### Statistical method of analysis

Values are expressed as mean ± standard error of mean (S.E.M). The values were subjected to Analysis of Variance (ANOVA), as well as Duncan's multiple Range Test, to determine statistical significance. Values with p<0.05 compared against the control were considered significant.

## Results

The effect of two different doses of aqueous extract of *Capsicum frutescens* and *Morinda lucida* is as shown in table 1. After the induction of pyrexia, the control rats remained hyperthermic throughout the duration of the experiment. The temperature of the rats treated with the reference drug decreased, 60 minutes post-administration. After 90 minutes, the temperature increased a little (though less than the pyretic temperature). By 120 minutes however, the temperature returned to normal. The two doses of both extracts also showed a reduction in rectal temperature similar to the pattern observed in the reference drug. Overall, however, all the doses reduced the rectal temperature to normal after 120 minutes. The results also showed that aqueous extracts of both *C. frutescens* and *M. lucida* leaves significantly (p<0.05) reduced the rectal temperature of the rats, especially after 120 minutes oral administration, compared to the pyretic state of rats in the control group.

**Table 1:** Antipyretic effects of indomethacin, extracts of *M. lucida* leaves and fruits of *C. frutescens*

Treatment	Doses (mg/kg)	Temperature (°C) before yeast	Temperature after 17 hours (°C)	Post extract/drug temperature (°C)		

				60 minutes	90 minutes	120 minutes
Control	10	37.52 ± 0.17^b^	38.32 ± 0.74^c^	38.12 ± 0.10^a^	38.10+0.12^a^	36.70+0.38^b^
Indometh.	5	36.72 ± 0.24^a,b^	37.06 ± 0.30^a,b^	36.88 ± 0.29^a^	37.04+0.33^a^	36.70±0.38^a,b^
***C.frutescens***	100	37.04 ± 0.34^a,b^	37.88 ± 0.15^c^	36.98 ± 0.36^a^*	37.08±0.29^a^*	36.66±0.26^a,b^
***C.frutescens***	200	36.74 ± 0.33^a,b^	38.82 ± 0.22^a^	36.96±0.27^a^*	36.78±0.37^a^*	36.42±0.45^a^
***M. lucida***	100	36.56 ± 0.22^a^	37.64 ± 0.14^b^	36.92±0.12^a^*	36.54±0.19^a^*	36.58±0.18^a^*
***M. lucida***	200	36.24 ± 0.30^a^	37.42 ± 0.22^a,b^	36.22±0.67^a^	36.68±0.39^a^	36.10±0.33^a^

Each value is a mean of five rats ± the Standard Error of Mean, (S. E. M).

Each value shown is for p<0.05

Different letters in a row shows that the figures are significantly different at P<0.05

* P<0.05 (as compared within each group)

Also, there is an apparent disparity in the potency of the two extracts in all the treatments, with the higher dose (200 mg/kg) of *M. lucida* showing the most apparent effect (Table 1); this was not however significant when they were subjected to statistical analysis. Also, the antipyretic effects of both extracts followed the same pattern, and were most significantly pronounced (p<0.05) after 120 minutes. The observed pattern is similar to that of indomethacin, a standard reference drug.

## Discussion

This present study evaluated the antipyretic activities of aqueous extract of fruits of *Capsicum frutescens* and *Morinda lucida* leaves, as well as their potency. Brewer's yeast, a well-documented febrile agent 27 was used to induce pyrexia. Brewer's yeast is an exogenous pyrogen, and, thus, has to stimulate endogenous pyrogens to induce hyperpyrexia^28^. Brewer's yeast induces both TNF-α and prostaglandin synthesis, especially PGE2. It also interacts with polymorphonuclear leucocytes to produce a polypeptide leukocyte pyrogen (endogenous pyrogen) 28–29. Leukocyte pyrogen is also known as cytokines. It stimulates the pre-optic region of the anterior hypothalamus with cells of the organum vasculascum, laminae terminalis to secrete PGE2 which in turn stimulates the hypothalamic thermostat to adjust the body temperature to a higher set point (above 37°C) and this result in pyrexia. Aqueous extract of *Morinda lucida*, natively called ‘oruwo’ is believed locally to possess effective antipyretic properties. *Capsicum frutescens*, ‘ata wewe’ or ‘ata ijosi’ is usually used traditionally as part of decoctions and is also believed to act as an antipyretic agent in the decoctions 9. Fever usually accompanies most infections, hence the quest for antipyretic herbs. This study seeks to lend scientific credence to the use of the extracts as antipyretics, and evaluate their potency using standard laboratory procedures. From the results, aqueous extract of both *C. frutescens* and *M. lucida* at the doses of 100-200 mg/kg body weight possess antipyretic activities in a time dependent manner. The antipyretic activities of the aqueous extract of *C. frutescens* and *M. lucida* may be attributed to inhibition of prostaglandin release by blocking enzymes like PGE2 synthase, cyclooxygenase-2 (COX-2) and phospholipase A2. Prostaglandins belong to a group of signaling molecules called eicosanoids. They exert complex control over various body mechanisms, and result from a common precursor, arachidonic acid in a pathway called the arachidonic acid pathway. PGE2 synthase, cyclooxygenase-2 (COX-2) and phospholipase A2 are all enzymes that mediate this pathway. These enzymes mediate the arachidonic pathway by bringing about prostaglandin (PGE2) release. The antipyretic activity of the aqueous extract of *C. frutescens* and *M. lucida* may be attributed to inhibition of prostaglandin release by blocking these enzymes. It could also be as a result of the inhibition of the synthesis of some cytokines like tumour necrosis factor, Interleukin-6 and Interleukin-8. Even though the 200 mg/kg dose of *M. lucida*, after 120 minutes apparently lowered the temperature more than all the other groups (table 3), this was not significant when subjected to statistical analysis. This suggests a similarity in the potency of the two extracts and that the extracts are effective, even at minimal dose. It is interesting to note that a fruit with such ‘biting sensation’ like *C. frutescens* will possess such antipyretic activities. However, further studies will focus on elucidating the phytoconstituents of these plants to ascertain the bioactive metabolites, with emphasis on the expression of TNF, PGE2, COX-2, and cytokines (IL-6 and 8) in pyretic animal models treated with these herbs.

## Conclusion

These findings justify the claim that aqueous extracts of Morinda lucida leaves and fruits of Capsicum frutescens possess antipyretic properties, with similar potency. The biting sensation of Capsicum frutescens may explain why it is not usually used singly but in combination with other herbs, and the extracts might be exerting multiple activities when administered traditionally to treat malaria fever.
